# The 10^th^ Annual Symposium on Global Cancer Research: New Models for Global Cancer Research, Training, and Control

**DOI:** 10.1200/GO.22.00122

**Published:** 2022-05-05

**Authors:** Fatou Jallow, Maria Teresa Bourlon, Mishka Kohli Cira, Kalina Duncan, Linsey Eldridge, Erinma Elibe, Taylor Estes, Allison Frank, Patti Gravitt, Andrea Sabina Llera, Corrina Moucheraud, Rita Mulherkar, Webster Musonda, Javier Gordon Ogembo, Paul Pearlman, Sam Phiri, Sudha Sivaram, Mariana Stern, Satish Gopal

**Affiliations:** ^1^ Center for Global Health, US National Cancer Institute, Rockville, MD; ^2^ Academic Global Oncology Task Force, American Society of Clinical Oncology, Alexandria, VA; ^3^ Hematology and Oncology Department, Instituto Nacional de Ciencias Médicas y Nutrición Salvador Zubirán, Mexico City, Mexico; ^4^ Translational Genomics, Fundación Instituto Leloir-CONICET, Buenos Aires, Argentina; ^5^ Health Policy and Management, UCLA Fielding School of Public Health, UCLA Jonsson Comprehensive Cancer Center, UCLA Center for Health Policy Research, Los Angeles, CA; ^6^ American Association for Cancer Research, Philadelphia, PA; ^7^ Advanced Center for Treatment, Research and Education in Cancer, Navi Mumbai, India; ^8^ Department of Surgery, University Teaching Hospitals—Adult and Emergency Hospital, Ministry of Health, Lusaka, Zambia; ^9^ City of Hope Global Oncology and Diabetes Initiative, City of Hope Comprehensive Cancer Center, Duarte, CA; ^10^ Global Health Technology, Center for Global Health, US National Cancer Institute, Rockville, MD; ^11^ Partners in Hope Malawi, Lilongwe, Malawi; ^12^ Department of Medicine, University of North Carolina, Chapel Hill, NC; ^13^ Department of Global Health, University of Washington, Seattle, WA; ^14^ Department of Public Health and Family Medicine, Kamuzu University of Health Sciences, Lilongwe, Malawi; ^15^ USC Norris Comprehensive Cancer Center, Los Angeles, CA; ^16^ Population and Public Health Sciences and Urology at the Keck School of Medicine, University of Southern California, Los Angeles, CA

As we enter the third year of the global COVID-19 pandemic and broader social justice movement, long-standing inequities in health are again receiving heightened attention. This includes cancer, which continues to place a growing burden on low- and middle-income countries (LMICs), where nearly 70% of cancer cases are estimated to occur by 2040.^1^ Global cancer research has not always sought nor included perspectives from communities most impacted by cancer, particularly those in LMICs. It is important, therefore, to create spaces within the global cancer research community which allow a diversity of voices to be heard. Informed by such diverse perspectives, it is then incumbent upon organizations working in this space to rethink our models for cancer research to expand our ability to meet a goal of more equitable and effective cancer prevention and control.

The National Cancer Institute (NCI) Annual Symposium on Global Cancer Research (ASGCR) seeks to increase visibility and prioritization for cancer research and control within the larger global health dialogue and strives for inclusive participation in alignment with the NCI Center for Global Health (CGH) core values.^2^ In 2013, the NCI/CGH, in partnership with the Consortium of Universities for Global Health (CUGH), established the ASGCR as a satellite meeting to the CUGH annual global health conference. The most recent ASGCR was held virtually on March 23-24, 2022, with a conference theme of *New Models for Global Cancer Research, Training, and Control*. This was the Symposium's 10th anniversary which included an inaugural Early Career Investigator Day (ECID) as a pre-Symposium scientific session on March 22, 2022. The ASGCR is led by the NCI/CGH in partnership with the CUGH, ASCO, American Association for Cancer Research and several NCI-Designated Cancer Centers. This year, NCI-Designated Cancer Centers from the Los Angeles area, where the CUGH conference was originally to be held, and their international collaborators, served on the Scientific Steering Committee. This included the USC Norris Comprehensive Cancer Center, United States, UCLA Jonsson Comprehensive Cancer Center, United States, City of Hope Comprehensive Cancer Center, United States, University Teaching Hospital, Ministry of Health, Republic of Zambia, Partners in Hope, Malawi, and Fundación Instituto Leloir-CONICET, Argentina.

The inaugural ASGCR ECID provided an opportunity for early-career scientists to engage with peers and invited experts, and to discuss cancer research and program development. One hundred forty early-career investigators representing 30 countries were to participate in the inaugural event. More than 65% represented LMICs and most registrants stated their primary interest as global cancer research program development or grant writing skill development. The topics covered included competencies in global cancer research, forming equitable collaborations, supporting career advancement, and perspectives from the National Institutes of Health awardees and collaborators. Breakout room discussions allowed early career investigators to interact in a smaller setting about the need for equitable collaborations and peer-peer mentoring.

ASGCR included two scientific panels on topics identified by the Scientific Program Committee as timely and relevant to the global cancer research and control community. These panels included cross-cultural and trans-disciplinary experts who participated in interactive facilitated discussions on practical solutions to cancer control challenges and creative methods for evidence synthesis and dissemination.

The first ASGCR scientific panel focused on systems thinking and its potential to use novel research methods to address complexity in a new way. Systems thinking has long been applied in many other disciplines and can provide a new paradigm to identify problems and design solutions in global cancer research.^3^ The panel emphasized the importance of applying systems approaches even when developing and initiating new studies to ensure contextually appropriate research translation that strongly considers and responds to the needs of local communities and patients, and then generates generalizable implementation knowledge that can be disseminated globally. They further elaborated on specific approaches to translate research into policy and bridge the translational gap using the appropriate tools within different regions.

The second scientific panel explored academic global cancer research collaborations, including challenging existing models, and exploring more equitable structures to support cancer research. The panel discussed global cancer research conduct, including mentorship and career tracks, research infrastructure, and knowledge generation. Special emphasis was placed on inequities in research collaborations and frequent misalignment between funding agencies and LMIC needs and priorities. Speakers also highlighted the constraints encountered by investigators based in LMICs, such as the need to self-fund research projects and a lack of protected time from clinical obligations.

In both panels, the speakers and audience engaged with Mentimeter live polling, allowing immediate feedback on the topics being discussed. Themes that emerged from this interactive discussion on both panels included: a need for more support—both financial and protected time—for early career scientists in LMICs, the importance of appropriate and equitable mentorship and training opportunities, and ensuring better alignment of priorities between funding agencies, high-income country investigators and institutions, and LMIC investigators and institutions.

Complementing the two scientific panels, 63 abstracts were accepted for poster presentation and publication in this special issue of ASCO's *JCO Global Oncology*, of which 10 were also selected for oral presentation during the Symposium. Both days of the Symposium included an interactive virtual poster session where abstract authors interacted with participants to share their scientific work and network. The abstracts published in this issue were submitted by authors representing 52 institutions from 17 countries, with research or programs conducted in 38 countries. Abstracts spanned the cancer continuum, with the majority focused on (1) cancer control, survivorship, and outcomes research, or (2) early detection, diagnosis, or prognosis.

At the close of the meeting, the Scientific Steering Committee presented the 2022 Rachel Pearline Award to Dr C.S. Pramesh, Director of the Tata Memorial Hospital and Professor and Head of Thoracic Surgery at the Tata Memorial Center in Mumbai, India, in recognition of his commitment and efforts to reduce inequities in cancer care and make high-quality cancer treatment accessible to all geographic regions and strata of society within India. Accepting this award, Dr Pramesh delivered the closing keynote address at the 2022 ASGCR.

As the global cancer research and control field strives for greater equity, new research partnership models are required that address historical assumptions and injustices.^4^ Bidirectional training of diverse early-career investigators from LMICs and HICs must be prioritized. Applying systems thinking to solve global cancer control challenges and revising academic models to achieve more sustainable global cancer research and control can create a more equitable field and ensure that cancer research anywhere benefits people everywhere. The NCI/CGH and partners will continue to use the ASGCR and newly established ECID as platforms to listen, amplify, and respond to voices from the global cancer research and control community. Through our partnership with ASCO, we are grateful to feature the 2022 ASGCR scientific abstract authors sharing their science in this *JCO Global Oncology* special supplement.
